# GestuRe and ACtion Exemplar (GRACE) video database: stimuli for research on manners of human locomotion and iconic gestures

**DOI:** 10.3758/s13428-017-0942-2

**Published:** 2017-09-15

**Authors:** Suzanne Aussems, Natasha Kwok, Sotaro Kita

**Affiliations:** 1 Department of Psychology, University of Warwick, CV4 7AL Coventry, UK; 2 P.L.A.I. Behaviour Consulting, Hong Kong, P.O. Box 11010, General Post Office, Hong Kong

**Keywords:** Action exemplars, Iconic gestures, Human locomotion manners, Video database, Stimuli set

## Abstract

Human locomotion is a fundamental class of events, and manners of locomotion (e.g., how the limbs are used to achieve a change of location) are commonly encoded in language and gesture. To our knowledge, there is no openly accessible database containing normed human locomotion stimuli. Therefore, we introduce the GestuRe and ACtion Exemplar (GRACE) video database, which contains 676 videos of actors performing novel manners of human locomotion (i.e., moving from one location to another in an unusual manner) and videos of a female actor producing iconic gestures that represent these actions. The usefulness of the database was demonstrated across four norming experiments. First, our database contains clear matches and mismatches between iconic gesture videos and action videos. Second, the male actors and female actors whose action videos matched the gestures in the best possible way, perform the same actions in very similar manners and different actions in highly distinct manners. Third, all the actions in the database are distinct from each other. Fourth, adult native English speakers were unable to describe the 26 different actions concisely, indicating that the actions are unusual. This normed stimuli set is useful for experimental psychologists working in the language, gesture, visual perception, categorization, memory, and other related domains.

**Electronic supplementary material:**

The online version of this article (10.3758/s13428-017-0942-2) contains supplementary material, which is available to authorized users.

## Introduction

Human locomotion (e.g., movement of the human limbs to change location) is a topic widely studied in the field of experimental psychology. For instance, expressions of human locomotion have been studied in spoken language (e.g., Malt et al. 2008; Slobin et al. 2014; Malt et al. 2014), written language (e.g., Slobin 2004, 2006), sign language (e.g., Supalla 2009; Slobin & Hoiting 1994), and gesture (e.g., Özyürek 1999; Kita 2003; Özçalışkan 2016). Also, in many word learning experiments, researchers teach children verbs for novel manners of human locomotion (e.g., Mumford 2014; Mumford & Kita 2014; Imai et al. 2008; Scott & Fisher 2012). In memory experiments, locomotion stimuli are often used to study visual memory of agents and their actions (e.g., Wood 2012). In categorization experiments, human locomotion is used to study, inter alia, how children perceptually categorize manners of locomotion (e.g., Salkind et al. 2003; Salkind et al. 2005; Pulverman et al. 2006).

Particularly in studies on verb learning, human locomotion stimuli are often used along with iconic gestures. Iconic gestures (McNeill 1992) represent actions, motions or attributes associated with people, animals, or objects (e.g., wiggling the index and middle fingers to represent a person walking; tracing a shape). Researchers have investigated whether novel verb meanings are shaped by iconic gestures that are shown when the verb is taught (e.g., Spencer et al. 2009; Goodrich & Hudson Kam 2009; Mumford 2014; Mumford & Kita 2014).

Developing human locomotion stimuli can be very laborious. Nevertheless, most researchers develop such stimuli solely for the purpose of their own research. As a consequence, there is no openly accessible video database containing manners of human locomotion and iconic gestures that represent these manners.

## Current Research

### Contents of the GRACE Video Database

**Fig. 1:**
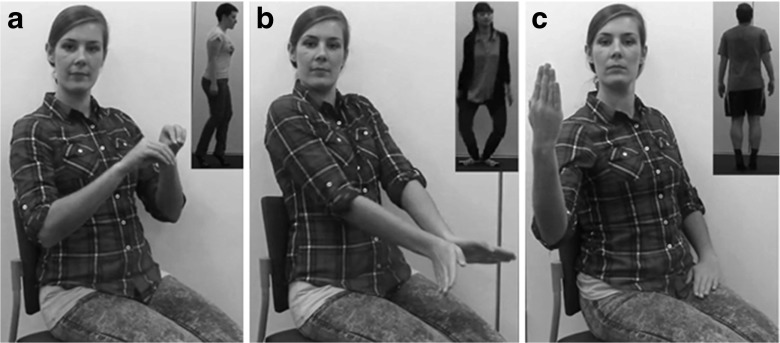
Three panels (A, B, and C) with cropped stills of videos in which a female actor gestures iconically to represent the manners of human locomotion performed by actors in the upper right corners of the panels. In the actual norming study, the action video and the gesture video had the same size and were presented side-by-side. Gestures and actions are included in separate video files in the database. From left to right the panels show the following gesture videos: “00F_scurrying.mp4”, “00F_mermaiding.mp4”, and “00F_twisting.mp4”, and action videos: “01F_scurrying.mp4”, “09F_mermaiding.mp4”, “01M_twisting.mp4”

We developed and normed the GestuRe and Action Exemplar (GRACE) video database, which includes 676 videos of 26 actors (13 males, 13 females) performing 26 *novel* manners of human locomotion (i.e. moving from one location to another in an unusual manner), and 26 videos of a female actor who produces iconic gestures that represent these manners. Figure 1 presents three examples of the gestures and the corresponding manners of locomotion (in the upper right corner of each panel). The gesturing hands represent the actor’s feet (panel A), the actor’s legs (panel B), and the actor’s whole body (panel C).

The GRACE video database is openly available from the Warwick Research Archive Portal at nAlong with the 702 video files, we have made the raw data from our norming studies available and the Python scripts that we used to process the data. We also included a manual that contains guidelines on how to use the GRACE video database.

### Norming the GRACE Video Database

In this section, we identify and motivate four essential requirements for the type of stimuli in the GRACE video database. These requirements guided the design of our norming studies to assure its usefulness for experimental psychologists. The GRACE video database is particularly useful for researchers who need unusual human locomotion stimuli to study language and gesture, memory, and categorization. Below, we discuss the implications of each norming study in the context of these research areas.

First, the GRACE video database includes videos that were normed for the degree of match between action pairs and matching and mismatching iconic gestures. Many experiments in developmental psychology use two-way forced choice tasks. In such tasks, pairing actions that would appear as two choices is important. The design of our first norming experiment is motivated by this future use. Also, pairing actions made data collection for this study more manageable; if we did not pair, participants would have to rate a large number of action-gesture combinations that make “mismatches”. Action pairs with matching and mismatching gestures could be used in experiments with a two-way forced choice task in which one of the actions is congruent with gesture, but the other is incongruent. This is useful for research on word learning with the help of iconic gestures (e.g., Mumford & Kita 2014; Mumford 2014; Özçalışkan et al. 2014; Goodrich & Hudson Kam 2009), the intake of information conveyed by gesture and speech (e.g., McNeill et al. 1994; Cassell 1999; Özyürek et al. 2007), and memory recall for sentences with the help of gesture (e.g., Feyereisen 2006; Madan & Singhal 2012). Furthermore, these stimuli are useful for studies on processing gesture-speech combinations, in which researchers often manipulate the semantic relations between the two channels (i.e., gesture and speech match, mismatch, or complement each other) (e.g., McNeill et al. 1994; Cassell et al. 1999;Özyürek et al. 2007; Spencer et al. 2009). Thus, the first norming study tested matches and mismatches between iconic gestures and manners of human locomotion in all the 676 action videos. We then ran an algorithm over the norming scores to identify the best possible matches between iconic gestures and actions performed by male actors and female actors, separately. This led to a one-to-one assignment of male actors and female actors to action pairs. Action videos of the selected actors were used in the next norming study.

Second, GRACE contains videos that were normed for the similarity of the same actions within action pairs performed by male actors and female actors and the (dis)similarity of the different actions within action pairs performed by male actors and female actors. Researchers who introduce an actor-change in their experimental task (e.g., to test actor memory or verb generalization) often do this by changing between male actors and female actors, as they have naturally distinct appearances (e.g., Mumford 2014). For instance, word learning studies that take an exemplar-based approach could use videos that show different actors performing the same actions and the same actors performing different actions (e.g., Maguire et al. 2002; Maguire et al. 2008; Scott & Fisher 2012). Videos that show different actors moving in the same manner could also be useful for creating generalization tasks to test people’s understanding of locomotion verbs (e.g., Imai et al. 2008), and recognition tasks and change-detection tasks to test their memory of actors (e.g., Imai et al. 2005; Wood 2008). In all these tasks it is important that the manner of human locomotion is similar across the actor-change. Thus, the second norming study tested how similar male actors and female actors perform the same actions within action pairs, and how distinct each male actor and female actor performs the two different actions within action pairs. All actions that are included in the database were normed in this study, but participants rated only the videos of male actors and female actors who were assigned to an action pair because their performance matched corresponding gestures very well in the first norming study.

Third, GRACE includes 26 actions which were normed for how distinct they are compared to every other action in the database. In this norming study, we let go of the notion of action pairs to obtain a measure of distinctiveness for all the actions in the database. There are three advantages of using this approach. First, norming the distinctiveness between all 26 actions is useful for studies on the ways in which people can categorize various semantic components of motion verbs such as figure (e.g., the man, the woman, Pulverman *et al*. 2006) and manner (e.g., Salkind 2003; Salkind et al. 2005). Second, such norms are useful for studies on infants’ ability to discriminate manners of motion (e.g., Pulverman et al. 2008; Pulverman et al. 2013), which use change-detection tasks with more than two options (e.g., four actions presented to participants on each quadrant). Third, the manners of locomotion that are shown to one participant need to be highly distinctive from each other to avoid confusion in any given task. For example, if a participant is taught a novel label for a locomotion manner in a word learning task, then this manner should be distinct from all manners that are subsequently labeled to avoid a bias in test performance. Therefore, the third norming study tested the similarity between all combinations of actions to obtain a measure of distinctiveness for each action in the database. In this norming study, human raters were presented with a subset of the videos from the database, in which each video showed one of the 26 actions performed by either a male or female actor.

Finally, the 26 actions in the GRACE video database were normed for how accurately and concisely they can be described by adult native English speakers. We asked whether the English language contains existing single-word or multi-word labels for the actions, which we used as a measure of how unusual the actions are. It is important that the stimuli are unusual to ensure that a given task performance occurs as a function of an experimental manipulation and not as a consequence of participants being familiar with the stimuli prior to the task. This is important for language research: if a participant already knows a label for an action action that is labeled in a word learning task, then this may cause a bias in test performance. It is also important for memory research: if people commonly perform these actions in real life, then this may cause a bias in test performance. Therefore, the fourth experiment assessed how accurately and concisely each action can be described by adult native speakers of English. Participants described the 26 actions in the database based on the same set of videos as in the third norming study.

### General Methods for Developing the GRACE Video Database

The GRACE video database originated in work by Mumford and Kita (2014) and Mumford (2014), who developed 14 unusual manners of human locomotion and iconic gestures representing these manners. GRACE includes these 14 manners and 12 additional manners of human locomotion and corresponding iconic gestures, resulting in a total of 26 manners and gestures.

#### Action Videos

We recruited 13 male actors between 22–40 years old (*M* = 27.00, *SD* = 4.98) and 13 female actors between 20–42 years old (*M* = 27.08, *SD* = 6.36). The national origin of the actors varied from British, Czech, Japanese, Polish, Dutch, Indian, Irish, German, Canadian, Nigerian, Mauritian, Bulgarian, Pakistani, Singaporean, Malaysian to Chinese. All actors were educated to the university degree level.

Actors participated in individual recording sessions. They were instructed to keep their arms and hands by their side when performing the actions, because we needed the hand gestures of the female actor to unambiguously represent the actors’ feet, leg, and body movements. Actors were also required to carry out each action as an ongoing motion without any breaks.

Prior to recording each action, actors watched an example video of a model. The videos of the model were not included in the database so that all actors shared the same reference point when performing the actions. Subsequently, the actors were required to move across the length of a scene in the same manner as the model. The starting point and the ending point were marked on the floor just outside the camera view. Each action was recorded at least twice from a distance of approximately 4.5 meters. If actors struggled with one of the actions, the researcher showed them their last recorded video and practised the movement with them repeatedly until they were ready to record again. Every recording session lasted approximately 1 hour. Informed written consent was obtained at the end of each recording session.

#### Gesture Videos

Hand gestures of a female actor were recorded from a distance of approximately 1.5 meters. This actor watched the video recordings of the model performing an action prior to recording the gesture that was designed to match this action. Gestures were designed by the researchers based on the definition of iconic gestures by McNeill (1992) so that the form of gesture resembled the referent action.

Specifically, all gestures iconically represented the body part that was most prominent for each movement (i.e., feet, legs, or whole body), its dynamic shape, and the rate at which the movement was carried out. Gestures representing the whole body were performed with the right hand. Gestures representing the legs were performed by both hands, where the right hand represented the right leg and the left hand represented the left leg. Gestures representing the feet were performed with the fingers, where the right hand fingers matched the right foot and the left hand fingers matched the left foot.

### Apparatus

Videos were recorded using a Canon Legria HFR56 camera with autofocus in a room with controlled light settings. Recordings were muted, cut, optimized for HTML, and converted to MP4 files of 640 × 480 pixels using *avconv* on Linux. The total size of the GRACE video database is 185 mega-bytes.

## Experiment 1

The first experiment tested the degree of match (and mismatch) between iconic gestures and manners of human locomotion. During the development of the database, 26 iconic gestures were created that matched each action. A mismatch between iconic gestures and actions was set up in the following way. Every action was paired up with another action from the set to create 13 action pairs (see Table 1). We then showed participants each action with a matching iconic gesture, but also with the iconic gesture that was created for the other action in the action pair as a mismatching iconic gesture. Participants rated these matches and mismatches on a seven-point scale.

**Table 1:** Twenty-six manners of human locomotion organized in action pairs

Pair	Action *a*	Still frame	Action *b*	Still frame
1.	bowing		skating	
2.	wobbling		marching	
3.	mermaiding		overstepping	
4.	creeping		crisscrossing	
5.	turning		hopscotching	
6.	swinging		skipping	
7.	jumping		crossing	
8.	dropping		folding	
9.	twisting		stomping	
10.	trotting		hopping	
11.	flicking		dragging	
12.	grapevining		shuffling	
13.	groining		scurrying	

Still frames are taken from the videos of the male actor whose videos file names start with “08M_”. Short-hand action labels are used to refer to the manners of locomotion and follow after the underscore in the file names of the database (e.g., “08M_bowing.mp4”, “08M_skating.mp4”)

We predicted that match ratings for matching iconic gestures and actions would be higher than match ratings for mismatching iconic gestures and actions. Additionally, we predicted that matches would be rated higher than the neutral score on a seven-point scale and that mismatches would be rated lower than the neutral score.

### Method

#### Participants

We recruited 301 individuals (183 males, 117 females) from the university’s online participant pool. Eight participants were excluded from further analyses because they indicated that the videos did not display, or run smoothly. The final participant sample included 293 individuals (179 females, 113 males) between 18–67 years old (*M* = 22.19, *SD* = 6.66). The majority of participants reported English as their native language (58.7%), followed by Asian languages (23.2%), and other Indo-European languages (18.1%). Participants automatically entered a lottery for an Amazon voucher upon completing the task.

#### Materials

**Fig. 2:**
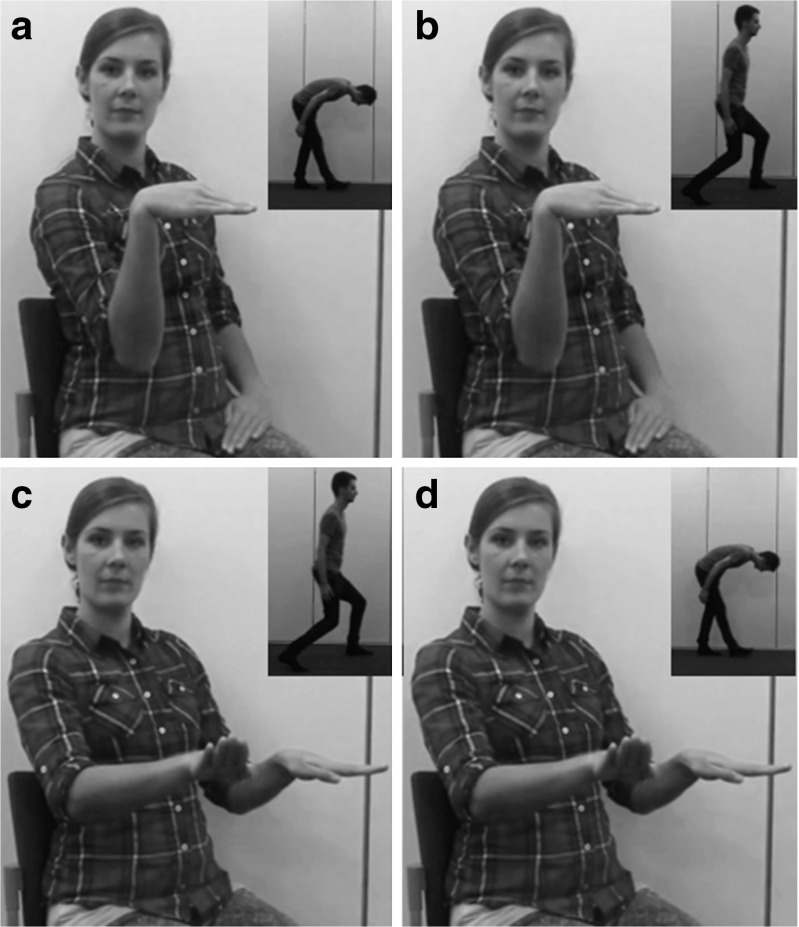
Four panels (A, B, C, and D) with cropped stills of videos in which a female actor gestures iconically to represent the actions of pair 1, as performed by a male actor in the upper right corners of the panels. Panels A shows a bowing gesture with a bowing movement (match), Panel B shows a bowing gesture with a skating movement (mismatch), Panel C shows a skating gesture with a skating movement (match), and Panel D shows a skating gesture with a bowing movement (mismatch). Gesture videos are “00F_bowing.mp4” (Panel A and B) and “00F_skating.mp4” (Panel C and D). Action videos are “06M_bowing” (Panel A and D) and “06M_skating” (Panel B and C)

We used videos of 26 manners of locomotion carried out by 26 actors (676 videos in total), and 26 videos of a female actor producing iconic gestures. Actions were organized in pairs (see Table 1) so that matches and mismatches between iconic gestures and actions could be created. Figure 2 shows the matches and mismatches between iconic gestures and actions for action pair 1. For instance, participants were shown *bowing* with a *bowing gesture* (Panel A), *bowing* with a *skating gesture* (Panel B), *skating* with a *skating gesture* (Panel C), and *skating* with a *bowing gesture* (Panel D).

We created 26 batches of videos to keep the length of the experiment reasonable. Each video batch contained videos of the 26 actions, but performed by different actors to ensure that all 676 action videos appeared in one of the batches. Each action video was combined with a matching and mismatching gesture video within a batch, which resulted in 52 trials. Each action video–gesture video combination was rated by on average 23 participants (range = 18 to 28).

#### Procedure

The experiment was set up in a web-based environment. Participants signed a digital consent form and were asked for demographic information. The instruction page showed participants a still frame of a gesture video and a still frame of an action video from the model as an example of a very good match. Participants were then shown two videos side-by-side, which started playing on loop automatically when a trial started. Participants were instructed to rate the match between the hand gesture of the female actor (left video) and the manner in which an actor moved (right video) on a seven-point scale, where 1 indicated a very bad match, 4 indicated neither a good nor a bad match, and 7 indicated a very good match. Participants were randomly assigned to one of the 26 batches and trials were randomly displayed for each participant. After they had seen all the trials, they were asked if all the videos ran smoothly, and if not, what type of problems had occurred.

#### Data Analysis

Using the *irr* package in the *R* software for statistical analyses (R Development Core Team 2011), we computed Kendall’s *W* (also known as Kendall’s coefficient of concordance) to assess agreement between participants who rated the same video batch. Kendall’s *W* is a non-parametric test statistic that takes into account the number of raters and the fact that the videos were rated on an ordinal scale. Its coefficient ranges from 0 (no agreement) to 1 (complete agreement).

We used non-parametric tests to analyze the ratings for matches and mismatches between iconic gestures and actions, because these ratings were not normally distributed. The *R* script containing the basic code for all analyses reported in this paper is uploaded as supplementary material.

#### The Hungarian Algorithm

We split the data based on the gender of the actors, because our aim is to identify the best possible match between iconic gestures and action pairs carried out by male actors and by female actors. The matrix containing average ratings for female actors was subjected to the Hungarian algorithm (Kuhn and Yaw 1955; Kuhn 1956) to find the most profitable (here best overall *match* between gestures and actions) assignment of 13 female actors to 13 action pairs (each actor can be assigned to only one action pair). In order to achieve a one-to-one assignment the matrix has to have the same number of rows and columns. The same procedure was carried out for the matrix containing average ratings for 13 male actors.

The Hungarian method (Kuhn and Yaw 1955; Kuhn 1956) finds an optimal assignment for a given *n* ⋅ *n* matrix in the following way. Suppose we have *n* action pairs to which we want to assign *n* actors on a one-to-one basis. The average ratings are the profit of assigning each actor to each action pair. We wish to find an optimal assignment which maximizes the total profit.

Let *P*_*i*,*j*_ be the profit of assigning an *i* th actor to the *j* th action pair. We define the profit matrix to be the *n* ⋅ *n* matrix:

1 $$ P = \left[\begin{array}{llll} P_{1,1} & P_{1,2} & {\cdots} & P_{1,n} \\ P_{2,1} & P_{2,2} & {\cdots} & P_{2,n} \\ {\vdots} & {\vdots} & & {\vdots} \\ P_{n,1} & P_{n,2} & {\cdots} & P_{n,n} \end{array}\right]. $$

An *assignment* is a set of *n* entry positions in the matrix, none of which lie in the same column or row. The sum of the *n* entries of an assignment is its profit. An assignment with the highest profit is called an optimal assignment. We implemented this algorithm in Python using the *Munkres* package. Our Python scripts are available from the Warwick Research Archive Portal at http://wrap.warwick.ac.uk/78493.

### Results and Discussion

#### Inter-Rater Reliability

Kendall’s *W* averaged over all 26 video batches was .72 (*SD* = 0.07) and ranged between .54 and .81. This coefficient was statistically significant for all batches (*p* < .001), indicating that participants were applying the same standards when rating the stimuli.

#### General Findings

**Fig. 3:**
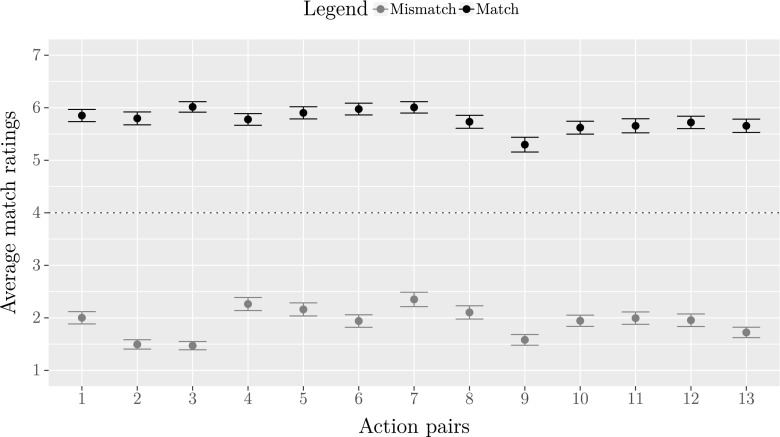
Average ratings for the degree of match between matching and mismatching iconic gestures and actions, organized by action pair. Error bars represent 95% confidence intervals of the means. Rating scores are averaged across all actors and represent the degree of match between iconic gestures and actions on a scale of 1 (“very bad match”) to 7 (“very good match”). The dotted line indicates the neutral score of 4 on the seven-point scale

Figure 3 displays the average ratings for the degree of match between iconic gestures and actions. Black dots represent average ratings for matches between iconic gestures and grey dots represent average ratings for mismatches between iconic gestures and actions. The 95% confidence intervals for both match and mismatch ratings are generally very narrow, indicating strong agreement among the participants.

We asked whether ratings differed between match and mismatch combinations of iconic gestures and actions. Ratings for matches and mismatches between iconic gestures and actions were averaged across all action pairs for each participant. A Wilcoxon rank sum test demonstrated that the median of average match ratings (*Mdn* = 5.92) was significantly higher than the median of average mismatch ratings (*Mdn* = 1.77), *W* = 316.5, *p* < .001, 95% CI of the difference [ −4.12, −3.88].

Furthermore, we compared the averaged ratings for matches and mismatches across action pairs against the neutral score on our seven-point scale. A Wilcoxon signed rank test indicated that the median of average match ratings was significantly higher than a neutral score of 4, *W* = 42638, *p* < .001, 95% CI of the median [5.77, 5.92]. In contrast, the median of average mismatch ratings was significantly lower than a neutral score of 4, *W* = 137, *p* < .001, 95% CI of the median [1.75, 1.92]. Thus, matching iconic gestures and actions were rated as good matches and mismatching iconic gestures and actions were rated as bad matches.

The 95% confidence intervals of the means in Fig. 3 clearly demonstrate that there is some variability between action pairs. When we compared the median of the averaged match and mismatch ratings for every action pair against a neutral score of 4, Wilcoxon signed rank tests revealed that matches and mismatches for all action pairs differed significantly from the neutral score (*p* < .001 for all comparisons).

#### Assigning Actors to Action Pairs

The Hungarian Algorithm optimally assigned 13 female actors to 13 action pairs, and did the same for 13 male actors. The Algorithm used “profit” matrices for actors and action pairs, created in the following way (one matrix for female actors, and another one for male actors). For each action performed by each actor, 10–14 participants rated the match between each action and a matching gesture. The ratings were averaged across participants, and then the two average ratings for actions that comprise an action pair were averaged again to create a “profit” for the action pair and actor.

For females, the algorithm selected the female actor with the highest match rating for an action pair eight times, the female with the second highest match rating for an action pair four times, and the female with the fourth highest match rating for an action pair one time. As the 13 females were assigned to 13 action pairs, the highest possible profit that could have been achieved was 91 (13 × 7). The algorithm assigned female actors to action pairs with a total profit of 80.63 (88.6% of 91), with the lowest average match rating for an assigned actor being 5.56 out of 7 (see Fig. 6 in Appendix).

For males, the algorithm selected the male actor with the highest match rating for an action pair six times, the male with the second highest match rating for an action pair two times, the male with the third highest match rating two times, the male with the fourth highest match rating two times, and the male with the fifth highest match rating one time. The algorithm assigned male actors to action pairs with a total profit of 81.02 (89.0% of 91), with the lowest average match rating for an assigned actor being 5.64 out of 7 (see Fig. 7 in Appendix).

Experiment 1 provided norming scores for all the videos in the GRACE videos database. With these ratings we evaluated the match and mismatch between iconic gestures and actions within action pairs. Moreover, the Hungarian algorithm over these ratings optimally assigned male actors and female actors to action pairs, to maximize the overall degree of match between gestures and action pairs. These assignments will be used in subsequent experiments.

## Experiment 2

The second experiment tested whether the male actors and female actors who were assigned to an action pair based on Experiment 1 perform the same actions in similar manners and the two different actions in distinct manners. Participants rated the similarity between two action videos on a seven-point scale. These videos showed either the same actor performing two different actions, or two different actors (male vs. female) performing the same action.

We predicted that two actors performing the same action would be rated more similar than the same actor performing two different actions. Additionally, we predicted that two actors performing the same action would be rated more similar than the neutral score on a seven-point scale and the same actor performing a different action would be rated less similar than the neutral score.

### Method

#### Participants

We recruited 42 individuals (19 males, 22 females, and 1 would rather not say) from the university’s online participant pool. Two participants were excluded from further analyses because they indicated that the videos did not display, or run smoothly. The final participant sample included 40 individuals (20 females, 19 males, and 1 would rather not say) between 18–57 years old (*M* = 24.30, *SD* = 8.25). The majority of participants reported English as their native language (67.5%), followed by other Indo-European languages (22.5%), and Asian languages (10.0%). Participants automatically entered a lottery for an Amazon voucher upon completing the task.

#### Materials

We used videos of male actors and female actors, who were assigned to the action pairs based on Experiment 1. Trials included either two videos of the same actor (male or female) performing the two different actions in a pair, or two videos of two different actors performing the same actions in a pair (action *a* or action *b*). Thus, for each action pair we created four trials, resulting in a total of 52 trials (13 action pairs × 2 actor gender × 2 same or different action).

#### Counterbalancing

The left–right position of the action videos on each trial was counterbalanced across participants using two different versions of the experiment.

#### Procedure

The procedure of this online experiment was similar to Experiment 1. The instruction page showed two videos of the same action performed by a male actor and a female actor (who were not included in the database) as a “very similar” example. The instructions stated that participants should not proceed if they were unable to view the videos properly.

During the main task, participants saw two videos side-by-side and rated the similarity between two movements on a seven-point scale, where 1 indicated very dissimilar, 4 indicated neither similar nor dissimilar, and 7 indicated very similar. Both videos started playing on loop automatically when a trial commenced. Participants were randomly assigned to an experiment version and trials were displayed in a random order for each participant. After they had seen all the trials, they were asked if all the videos ran smoothly, and if not, what type of problems had occurred.

#### Data Analysis

The data were analyzed in the same way as in Experiment 1.

### Results and Discussion

#### Inter-Rater Reliability

A statistically significant Kendall’s *W* of .77 (*p* < .001) was computed for the similarity ratings, indicating that participants reached agreement when rating the stimuli.

#### General Findings

**Fig. 4:**
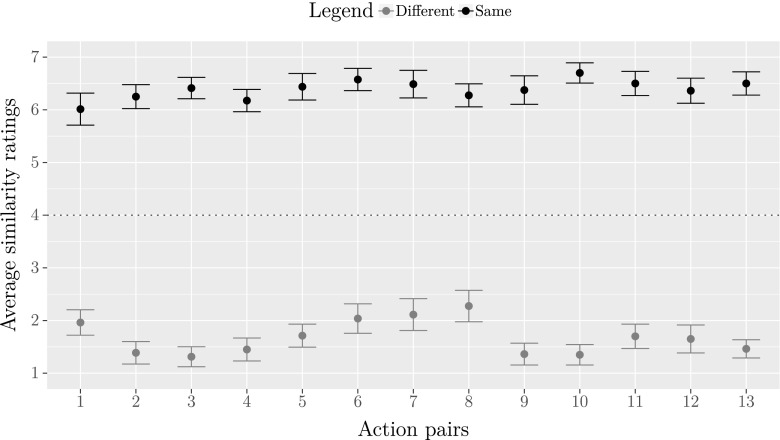
Average similarity ratings for actions within each action pair. Error bars represent 95% confidence intervals of the means. For each participant, ratings were averaged across the male actor and the female actor who were assigned to an action pair based on Experiment 1, separately for the same and different actions within each action pair. Rating scores represent the similarity between two actions, on a scale of 1 (“very dissimilar”) to 7 (“very similar”). The dotted line indicates the neutral score of 4 on the seven-point scale

Figure 4 displays the average similarity ratings for the same and different actions within each action pair, carried out by the male actors and female actors who were assigned to these action pairs based on Experiment 1. The 95% confidence intervals of the means for both the same and different actions are generally very narrow, indicating that participants reached agreement.

We asked whether ratings differ between different actors performing the same action and the same actors performing a different action. Ratings for the same actors performing two different actions and two different actors performing the same actions were averaged across action pairs for each participant. A Wilcoxon rank sum test demonstrated that the median of average ratings was significantly higher for two different actors performing the same action (*Mdn* = 6.62) than for the same actors performing a different action (*Mdn* = 1.48), *W* = 1.5, *p* < .001, 95% CI of the difference [ −5.19, −4.73].

We also predicted that two different actors performing the same action would be rated more similar than a neutral score of 4 and that the same actors performing a different action would be rated less similar than a neutral score of 4. Wilcoxon signed rank tests confirmed these predictions (different actors performing the same action *W* = 817, *p* < .001, 95% CI of the median [6.38, 6.65]; the same actors performing a different action, *W* = 820, *p* < .001, 95% CI of the median [1.40, 1.77]).

The 95% confidence intervals of the means in Fig. 4 evidently show that there appears to be some variability between action pairs. When we compared the median of averaged ratings for every action pair (for the same actor performing two different actions and two different actors performing the same actions) against a neutral score of 4, Wilcoxon signed rank tests revealed that ratings for all action pairs differed significantly from the neutral score (*p* < .001 for all comparisons). Overall, Experiment 2 thus shows that male actors and female actors, who were assigned to an action pair based on Experiment 1, perform the same actions in similar manners and different actions in distinct manners.

## Experiment 3

The third experiment tested how distinct the 26 actions are from every other action in the set. We used a subset of the video database, which included videos of the 26 actions carried out by the male or female actors who were assigned to an action pair based on Experiment 1. Participants rated the similarity between every combination of two action videos on a seven-point scale.

### Method

#### Participants

We recruited 225 individuals (88 males, 137 females) through the university’s online participant pool. Three participants were excluded from further analyses because they indicated that the videos did not display, or run smoothly. The final sample included 222 individuals (87 males, 135 females) between 18–73 years old (*M* = 24.04, *SD* = 8.69). The majority of participants reported English as their native language (55.9%), followed by other Indo-European languages (22.5%), and Asian languages (21.6%). Participants automatically entered a lottery for an Amazon voucher upon completing the task.

#### Materials

We used a set of 26 videos showing the 13 action pairs. For each action pair, we randomly determined whether each action was performed by the male or female actor that was assigned to that pair based on Experiment 1. If the male actor was selected for one action of the action pair, then the female actor was automatically selected for the other action of the action pair, and vice versa. Thus, 13 videos showed a male actor and 13 videos showed a female actor.

**Table 2:** Similarity rating matrix with averages (above the diagonal line of black squares) and standard deviations (below the diagonal line of black squares) for every combination of two action videos

	1a	1b	2a	2b	3a	3b	4a	4b	5a	5b	6a	6b	7a	7b	8a	8b	9a	9b	10a	10b	11a	11b	12a	12b	13a	13b
1a	■	2.38	4.23	1.43	1.57	2.00	2.68	1.48	1.10	1.95	1.38	2.23	1.95	1.86	3.67	1.74	2.23	1.89	1.43	1.62	1.48	2.19	1.26	3.95	2.19	2.24
1b	1.28	■	2.29	1.95	2.09	3.33	4.86	1.81	2.16	3.24	2.59	2.10	3.90	3.00	4.89	3.05	2.11	3.18	2.52	1.57	2.19	5.53	1.76	3.81	4.10	1.95
2a	1.57	1.35	■	1.24	1.71	1.38	1.95	1.38	1.48	2.33	1.57	2.58	1.62	1.58	2.38	1.52	4.11	1.90	1.43	1.48	1.62	1.57	2.36	2.43	1.84	2.90
2b	0.68	1.07	0.44	■	1.38	5.14	3.33	1.62	1.38	2.05	4.05	4.00	2.58	3.43	1.81	4.14	1.24	3.67	2.86	1.57	4.84	2.76	1.29	2.32	3.62	1.68
3a	0.87	1.27	1.27	0.67	■	1.19	1.43	3.33	4.27	2.84	1.16	2.14	2.14	1.43	2.00	1.57	3.38	2.16	1.90	5.42	1.67	1.29	3.19	1.62	1.67	2.29
3b	1.38	1.91	0.74	1.78	0.40	■	4.43	1.53	1.42	2.00	4.52	3.48	2.90	4.05	2.05	6.10	1.24	4.81	3.32	1.57	4.57	3.48	1.38	2.63	4.05	1.95
4a	1.80	1.74	1.09	1.85	0.68	1.33	■	2.05	1.81	2.57	2.48	3.41	3.33	4.41	5.43	4.00	1.19	3.90	3.10	1.67	3.16	4.95	1.68	3.57	3.95	2.67
4b	1.03	1.21	0.80	1.24	1.80	1.02	1.40	■	3.32	2.68	1.57	2.29	4.14	2.48	1.58	1.48	1.67	1.52	1.95	3.91	1.67	2.14	5.81	1.37	2.00	2.05
5a	0.30	1.21	0.75	1.12	1.32	0.84	1.21	1.83	■	2.82	1.57	1.62	2.19	1.43	1.79	1.38	3.76	1.29	1.81	4.05	1.43	1.71	2.14	1.24	1.76	1.62
5b	1.20	1.45	1.46	1.18	1.64	1.23	1.57	1.67	1.53	■	1.81	4.24	3.71	2.43	2.67	2.33	1.43	2.67	3.90	2.32	3.10	1.86	1.52	2.19	1.71	3.26
6a	0.74	1.50	1.03	1.40	0.50	1.50	1.44	0.75	1.40	1.03	■	2.24	1.90	3.76	1.73	3.84	1.38	2.58	1.90	1.42	2.76	3.27	1.67	1.81	4.33	1.62
6b	1.34	1.45	1.46	1.82	1.46	1.60	1.79	1.42	0.97	1.73	1.34	■	4.29	2.47	2.67	3.05	1.52	2.81	3.33	2.14	5.29	2.89	1.33	2.95	1.90	2.19
7a	1.32	1.84	0.86	1.54	1.15	1.61	1.32	1.28	1.03	1.78	1.22	1.62	■	3.10	2.71	2.19	1.33	3.71	3.91	2.05	4.16	3.29	2.42	3.23	2.73	3.16
7b	1.15	1.41	1.17	1.29	0.75	1.78	1.76	1.86	0.93	1.50	1.87	1.74	1.55	■	3.29	4.33	1.48	4.95	3.58	1.48	2.68	3.29	2.36	2.52	6.63	1.86
8a	1.98	1.79	1.24	0.98	1.20	1.28	1.54	0.69	1.40	1.59	0.98	1.71	1.62	1.79	■	2.62	1.33	3.05	2.38	1.91	1.90	3.84	1.90	3.43	2.90	2.71
8b	1.37	1.53	0.75	1.71	1.21	1.22	1.63	0.81	0.67	1.20	1.86	1.68	1.50	1.49	1.53	■	1.68	4.53	3.62	1.48	3.71	3.77	1.52	2.33	4.19	2.19
9a	1.69	1.33	1.94	0.54	1.80	0.70	0.68	1.28	1.97	0.87	0.80	0.90	0.73	0.81	0.66	0.89	■	1.59	1.71	2.10	1.76	1.32	2.43	1.76	1.62	3.43
9b	1.45	1.79	1.04	1.46	1.46	1.72	1.37	0.75	0.72	1.53	1.71	1.47	1.45	1.69	1.72	1.74	0.91	■	5.38	1.76	2.62	3.23	1.71	2.86	5.00	2.81
10a	0.68	1.40	1.16	1.61	1.04	1.38	1.41	1.40	1.17	1.81	1.22	1.91	1.93	1.68	1.16	1.72	0.90	0.86	■	1.38	4.86	2.10	1.90	1.95	3.42	4.76
10b	1.16	1.21	1.03	1.47	1.43	1.36	0.97	1.66	1.89	1.63	0.69	1.35	1.28	1.03	1.27	0.93	1.14	1.49	0.59	■	1.48	2.00	2.71	1.62	1.29	1.89
11a	0.98	1.29	1.28	1.50	0.97	1.12	1.50	1.15	0.81	1.73	1.45	1.15	2.11	1.52	1.04	1.82	1.41	1.36	1.64	0.81	■	2.43	1.53	2.81	2.50	2.33
11b	1.33	1.68	1.03	1.64	0.56	1.54	1.53	1.39	1.19	0.85	1.75	1.49	1.65	1.62	1.84	1.74	0.95	1.41	1.34	1.45	1.63	■	1.38	3.90	2.95	1.86
12a	0.65	1.22	1.73	0.64	1.54	0.59	1.06	1.17	1.31	0.93	1.06	0.58	1.46	1.68	1.14	1.03	1.29	1.10	1.34	1.49	1.02	0.59	■	2.52	2.36	2.19
12b	1.63	1.57	1.50	1.04	0.74	1.61	1.47	0.68	0.54	1.40	1.17	1.43	1.80	1.17	1.89	1.53	0.89	1.71	1.31	0.97	1.63	1.64	1.17	■	2.47	2.77
13a	1.08	1.61	1.21	1.66	1.32	1.60	1.86	1.10	1.30	0.96	1.53	1.14	1.42	0.60	1.76	1.94	1.02	1.64	1.71	0.78	1.26	1.60	1.68	1.02	■	1.52
13b	1.37	1.20	1.73	0.95	1.35	1.05	1.32	1.43	1.32	1.79	1.02	1.21	1.80	0.91	1.27	1.40	1.63	1.54	1.58	1.20	1.39	1.20	1.44	1.34	0.75	■

Columns and rows are labeled with numbers of the compared actions. *N* varies between 19 to 22 per cell. Average ratings are given above the diagonal line of black squares. Ratings represent the similarity between two actions, on a scale of 1 (“very dissimilar”) to 7 (“very similar”). Standard deviations are given below the diagonal line of black squares. See Table 1 for a more detailed description of what the action numbers (i.e., “1a”, “1b”, etc.) refer to

All possible combinations of two *different* action videos (26 × 25) were then divided over 26 video batches to keep the length of the experiment reasonable. We made sure that every action video appeared in each batch. Across batches each action video thus appeared with every other action video.

#### Procedure

The same procedure as Experiment 2 was used. Participants were presented with two action videos side-by-side, and rated the similarity between the actions on a seven-point scale, where 1 indicated very dissimilar, 4 indicated neither similar nor dissimilar, and 7 indicated very similar. Participants were randomly assigned to a video batch and trials were randomly displayed for each participant. After they had seen all the trials, they were asked if all the videos ran smoothly, and if not, what type of problems had occurred.

Participants were allowed to rate multiple video batches, because each batch presented participants with new combinations of action videos. We recorded 260 responses from 222 individuals. Every combination of two action videos was rated by on average 20 participants (range = 19 to 22).

#### Data Analysis

Inter-rater reliability was calculated in the same way as in Experiment 1-2. A similarity matrix was created by averaging the ratings over every combination of two actions.

### Results and Discussion

#### Inter-Rater Reliability

Kendall’s *W* averaged over all 25 video batches was .52 (*SD* = 0.12) and ranged between .27 and .68. This coefficient was statistically significant for all batches (*p* < .001), indicating that participants were applying the same standards when rating the stimuli.

#### General Findings

Table 2 shows similarity ratings for every combination of two actions. The average score was 2.56 (*SD* = 1.71) and ranged between 1.10 (*SD* = 0.30) for the combination of action 5a and 1a and 6.63 (*SD* = 0.60) for action 7b and 13a.

The distinctiveness of each action can be assessed by averaging similarity ratings between a given action and the other 25 actions: the smaller this average is, the more distinct the action is. According to this metric, action 9a (*M* = 1.94, *SD* = 1.40), 5a (*M* = 2.02, *SD* = 1.48), and 2a (*M* = 2.03, *SD* = 1.43) appear to be most distinct.

**Fig. 5:**
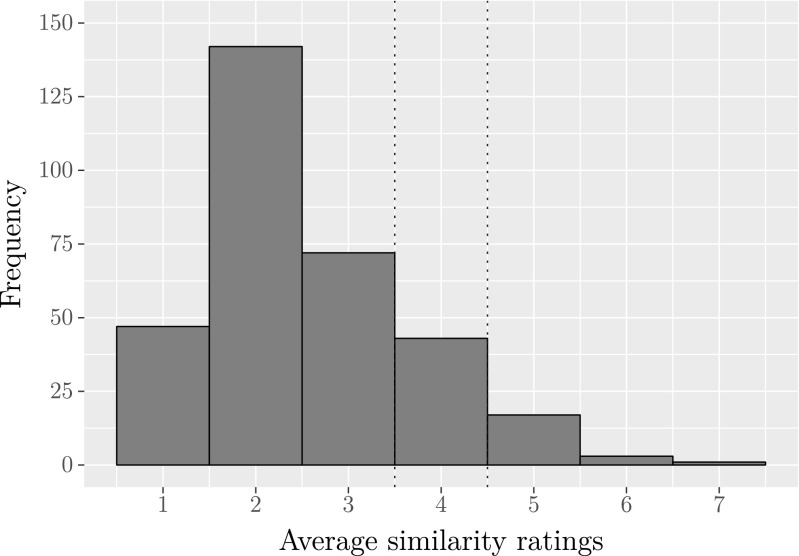
Frequency of average similarity ratings for all combinations of (different) actions in the database. *N* = 325 combinations of two different actions. Ratings represent the similarity between two actions, on a scale of 1 (“very dissimilar”) to 7 (“very similar”). The dotted lines mark the neutral score of 4 on the seven-point scale

**Fig. 6:**
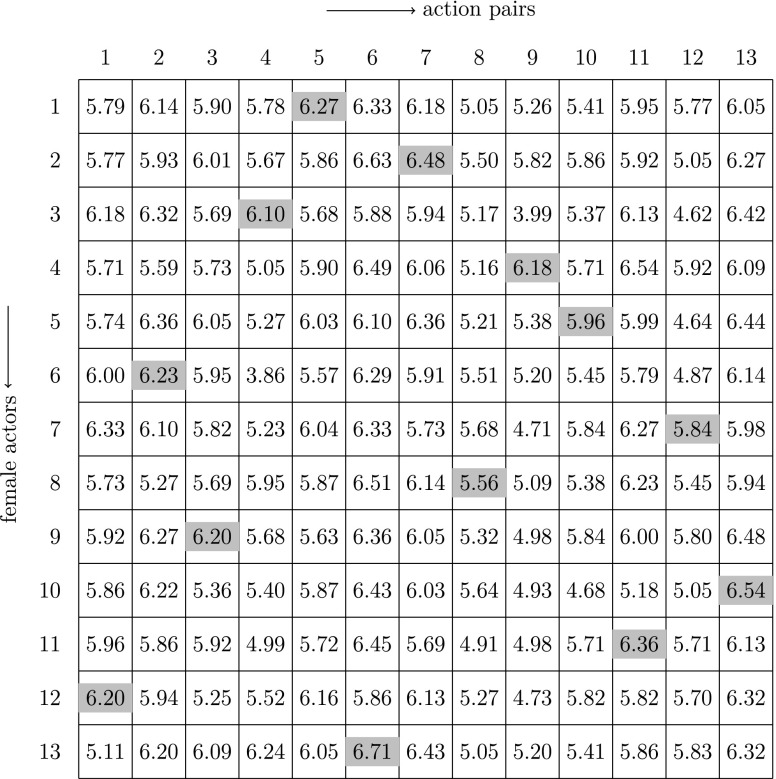
Profit matrix for action–gesture matches for females. Stimuli were rated on a seven-point scale, where 1 indicated a very bad match, 4 indicated neither a good nor a bad match, and 7 indicateda very good match. Ratings were averaged over each action pair and actor combination. Column numbers correspond to action pairs and row numbers correspond to the female actors in the database. Grey rectangles indicate the ratings that were selected by the Hungarian algorithm, which maximized the total profit of a one-to-one assignment of female actors to action pairs

**Fig. 7:**
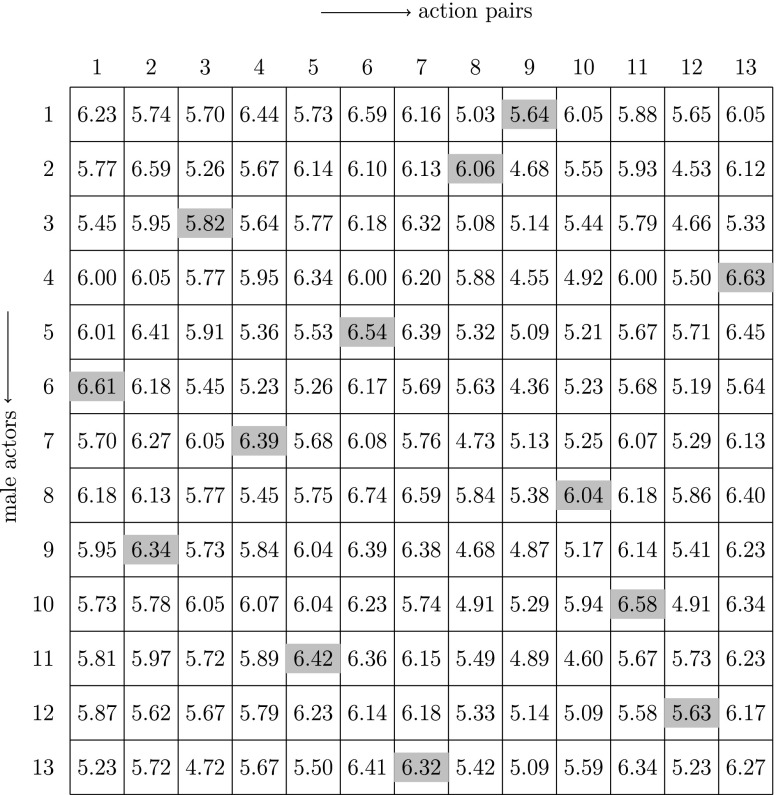
Profit matrix for action–gesture matches for males. Stimuli were rated on a seven-point scale, where 1 indicated a very bad match, 4 indicated neither a good nor a bad match, and 7 indicated a very good match. Ratings were averaged over each action pair and actor combination. Column numbers correspond to action pairs and row numbers correspond to the male actors in the database. Grey rectangles indicate the scores that were selected by the Hungarian algorithm, which maximized the total profit of a one-to-one assignment of male actors to action pairs

Figure 5 shows that most combinations of actions (80.6%) were rated–on average–on the left side of the seven-point scale (i.e. the area left side of the first dotted line), indicating that most actions are distinct from each other. The area between the two dotted lines covers the combinations of actions that participants rated neutrally (12.9%). Very few combinations of actions (6.5%) were rated–on average–on the right end of the seven-point scale (i.e. the area on the right side of the second dotted line), indicating that only some of the actions are similar to each other.

## Experiment 4

The fourth experiment assessed how accurately and concisely adult native English speakers can describe the actions in our database. This can also be used as the proxy measure for how unusual adult native English speakers find each action. If our participants find the actions unusual, then they should not converge on single-word or multi-word labels for the actions.

### Method

#### Participants

We recruited 28 native English speakers (10 males, 18 females) from the university’s online participant pool. One participant was excluded from further analyses because the videos did not display, or run smoothly. Three participants were excluded because they reported their first language to be something other than English. The final participant sample included 24 individuals (8 males, 16 males) between 18–48 years old (*M* = 22.92, *SD* = 6.43). Participants automatically entered a lottery for an Amazon voucher upon completing the task.

#### Materials

Experiment 4 used the same videos as Experiment 3.

#### Procedure

The experiment was set up in a web-based environment. Participants signed a digital consent form and were asked for demographic information. Prior to the main task, participants were shown a video of the model moving across the length of a scene. The instructions stated that every following video would also show an actor moving across the length of a scene, and that they had to describe the actor’s manner of movement as concise and accurate as possible. Participants were instructed to type an “X” to skip a trial in case they could not come up with a description for the movement. Participants were also asked not proceed if they were unable to view the video on the instruction page properly.

During the main task, a video started playing on loop automatically in the center of the screen on each trial. Participants were required to answer the question “Please describe the actor’s manner of movement as *concise* and *accurate* as possible:” using a text box below the video. Participants also rated the difficulty of coming up with a description on a seven-point scale, where 1 indicated very difficult, 4 indicated neither difficult nor easy, and 7 indicated very easy. Trials were randomly displayed for each participant, until participants had seen all actions. After they had completed all trials, they were asked if all the videos ran smoothly, and if not, what type of problems had occurred.

#### Data Analysis

Verbatim responses were spell-checked and converted to lowercase letters. The length of the descriptions was measured by counting the number of words separated by a blank space. Any punctuation (e.g., hyphens) did not count towards the number of words in a description.

We then annotated the content words in the descriptions using a Cambridge English dictionary. Nouns, main verbs, adjectives and adverbs are content words, which usually refer to some object, action, or characteristic of an event. Verbs, adjectives, and nouns (i.e., *rotate*, *rotating*, and *rotation*) that have the same root were coded as the same responses using the root of the word (i.e. *rotate*). Annotations could contain the same root more than once, but only unique roots counted towards the total number of content words in a description. For instance, one participant described action 11a with “*jump* forward and alternate your legs with each *jump* like a scissor movemenn, using the word “jump” first as verb and then as a noun. These two words have the same root and therefore only added a count of one to the total count of content words per description. Auxiliary verbs, pronouns, articles, and prepositions are grammatical words and were therefore not coded. Annotations were checked by an independent researcher.

We used two key statistics to evaluate the conciseness of the descriptions: the average number of unique roots per description and the number of descriptions that contained a single root. We computed the percentage of participants that mentioned the same root for each action to measure agreement among participants. Subsequently, these roots were ranked based on how many of the participants used them in their description and the three most used roots were reported for each action. Difficulty ratings were averaged over each action.

### Results and Discussion

Table 3 shows that participants provided quite lengthy descriptions for the actions (mean number of words per description: *M* = 6.99, *SD* = 5.71), ranging between 4.50 words for action 9b and 9.80 words for action 8b. On average, 4.68 (*SD* = 3.02) roots were annotated for the descriptions, ranging between 3.50 for action 9b and 10b and 6.15 for action 8b.

**Table 3:** Descriptive statistics of written descriptions for all 26 actions in the GRACE database

No.	Action	X	No. words	No. roots	Root 1	Root 2	Root 3	No. single	Difficulty
		(%)	*M* (*SD*)	*M* (*SD*)	(%)	(%)	(%)	roots (%)	*M* (*SD*)
1a.	bowing	0 (0.0)	8.04 (6.22)	5.29 (3.51)	bend (66.7)	walk (58.3)	forward (54.2)	1 (4.2)	4.58 (1.38)
1b.	skating	2 (8.3)	6.32 (4.24)	4.18 (2.30)	zigzag (25.0)	slide (25.0)	forward (25.0)	1 (4.2)	4.08 (1.79)
2a.	wobbling	1 (4.2)	8.43 (5.18)	5.48 (2.69)	body (62.5)	rotate (41.7)	upper (37.5)	1 (4.2)	3.67 (1.71)
2b.	marching	0 (0.0)	5.92 (3.60)	4.29 (2.18)	leg (66.7)	forward (41.7)	march (29.7)	3 (12.5)	5.29 (1.46)
3a.	mermaiding	0 (0.0)	7.71 (6.80)	4.96 (3.63)	side (79.2)	jump (58.3)	together (33.3)	0 (0.0)	5.04 (1.40)
3b.	overstepping	0 (0.0)	6.92 (4.60)	4.96 (2.72)	leg (54.2)	step (33.3)	forward (33.3)	1 (4.2)	4.33 (1.37)
4a.	creeping	0 (0.0)	5.29 (6.83)	3.58 (3.24)	walk (29.2)	forward (29.2)	slow (25.0)	5 (20.8)	4.50 (1.96)
4b.	crisscrossing	0 (0.0)	7.29 (5.22)	4.79 (2.64)	side (75.0)	cross (62.5)	leg (62.5)	1 (4.2)	3.92 (1.69)
5a.	turning	1 (4.2)	9.39 (8.54)	5.91 (3.99)	jump (79.2)	side (58.3)	turn (29.2)	0 (0.0)	3.96 (1.33)
5b.	hopscotching	2 (8.3)	7.27 (8.02)	4.64 (3.98)	hopscotch (50.0)	leg (33.3)	jump (29.2)	4 (16.7)	4.04 (1.78)
6a.	swinging	1 (4.2)	8.78 (5.38)	5.30 (2.67)	leg (70.8)	circle (50.0)	walk (33.3)	1 (4.2)	3.71 (1.52)
6b.	skipping	5 (20.8)	8.21 (8.49)	5.00 (4.18)	forward (45.8)	leg (37.5)	move (25.0)	2 (8.3)	2.67 (1.74)
7a.	jumping	0 (0.0)	7.04 (4.25)	4.71 (2.46)	jump (58.3)	forward (45.8)	leg (41.7)	1 (4.2)	3.96 (1.46)
7b.	crossing	2 (8.3)	8.09 (4.68)	5.41 (2.74)	cross (50.0)	leg (45.8)	walk (37.5)	0 (0.0)	3.54 (1.35)
8a.	dropping	1 (4.2)	6.61 (6.52)	4.83 (3.77)	walk (58.3)	squat (33.3)	crouch (29.2)	2 (8.3)	4.00 (1.47)
8b.	folding	4 (16.7)	9.80 (7.21)	6.15 (3.17)	leg (45.8)	walk (41.7)	step (33.3)	0 (0.0)	2.92 (1.79)
9a.	twisting	2 (8.3)	5.77 (6.64)	4.27 (2.81)	rotate (33.3)	degree (25.0)	side (20.8)	2 (8.3)	3.76 (1.69)
9b.	stomping	0 (0.0)	4.50 (3.20)	3.50 (2.02)	knee (50.0)	high (41.6)	stomp (33.3)	5 (20.8)	5.42 (1.32)
10a.	trotting	1 (4.2)	5.43 (3.64)	4.04 (1.89)	knee (62.5)	high (54.2)	forward (37.5)	2 (8.3)	2.67 (1.88)
10b.	hopping	0 (0.0)	4.79 (3.75)	3.50 (2.32)	side (87.5)	jump (58.3)	together (37.5)	1 (4.2)	6.08 (1.14)
11a.	flicking	2 (8.3)	5.73 (4.00)	4.00 (2.12)	leg (41.7)	forward (33.3)	quick (20.8)	1 (4.2)	3.50 (1.62)
11b.	dragging	1 (4.2)	7.17 (5.65)	5.17 (3.28)	forward (50.0)	leg (41.7)	step (37.5)	2 (8.3)	4.42 (1.74)
12a.	grapevining	1 (4.2)	6.48 (5.88)	4.48 (2.98)	cross (62.5)	side (58.3)	leg (50.0)	2 (8.3)	4.04 (1.23)
12b.	shuffling	0 (0.0)	5.83 (4.39)	3.71 (2.31)	walk (50.0)	forward (37.5)	lift (20.8)	3 (12.5)	5.42 (1.56)
13a.	groining	3 (12.5)	9.48 (6.30)	6.14 (3.45)	leg (54.2)	walk (29.2)	knee (29.2)	0 (0.0)	2.96 (1.68)
13b.	scurrying	0 (0.0)	5.38 (4.53)	3.92 (3.03)	tiptoe (58.3)	step (33.3)	fast (25.0)	3 (12.5)	5.36 (1.06)

*N* = 24. Columns left to right describe the action number (No.), short-hand action label (Action), number (%) of participants who were not able to describe the action (X), length of a description (No. words), number of content words in a description (No. roots), three most used content words for each action (Root 1–3), number (%) of descriptions that contained a single content word (No. single roots), and the rated difficulty of coming up with a description (Difficulty, 1 = very difficult and 7 = very easy)

Participants generally approached the task by describing the actions using main verbs and modified these verbs using adjectives, adverbs, directional phrases, and nouns that specified the part of the body that was most involved in the movement. For instance, one participant described action 10b as “turn sideways and simply jump sideways whilst keeping your feet together” and another participant described action 4a as “walking forward crouching slightly with knees bent”.

For the next analyses, we excluded 30 responses that stated an “X”, because this indicated that participants could not come up with a description. Participants who attempted to describe the actions used only a single content word in 7.4% of the cases. In most responses, participants thus used more than one content word to describe the actions.

Participants rated the difficulty of coming up with a description on average with 4.22 (*SD* = 1.74), suggesting that the task was neither difficult nor easy. Participants found action 6b and 10a (*M* = 2.67) most difficult to describe and action 10b (*M* = 6.08) easiest to describe.

Finally, we correlated the length of the descriptions (i.e. the number of words) and the number of roots per description with the difficulty ratings for the actions. Participants who found it more difficult to describe the actions provided longer descriptions *r*(595) = .10, *p* = .017, and used more content words, *r*(595) = .12, *p* = .003.

## General Discussion

We developed the GestuRe and Action Exemplar (GRACE) video database, which is publicly available from the Warwick Research Archive Portal at http://wrap.warwick.ac.uk/78493. The GRACE video database contains 676 videos of 26 novel manners of human locomotion performed by 13 male actors and 13 female actors (i.e. actors moving from one location to another in an unusual manner), and videos of a female actor producing iconic gestures that represent these manners.

Our first norming study demonstrates that GRACE contains gesture and action videos that can be combined to create clear matches and mismatches between iconic gestures and manners of human locomotion. Based on the findings of this first norming study, we assigned two actors (one male and one female) to a pair of actions to maximize the match between the iconic gestures and actions. Our second norming study shows that male actors and female actors who were assigned to an action pair perform the same actions in very similar manners and the different actions in highly distinct manners. Our third norming study indicates that the majority of actions are, in fact, highly distinct from all other actions in the database. Our fourth norming study demonstrates that adult native English speakers do not converge on accurate and concise linguistic expressions for the actions in the database, indicating that these manners of human locomotion are unusual.

This database is useful for experimental psychologists working on action and gesture in areas such as language processing, vocabulary development, visual perception, categorization, and memory. By making our video database publicly available to the research community, we set out to inspire researchers to norm our videos for their own studies. We invite these researchers to share these norms with us and other researchers so that we can upload these along with the GRACE video database through the Warwick Research Archive Portal.

### Electronic supplementary material

Below is the link to the electronic supplementary material.

(PDF 6.33 KB)
